# Nutritional Status of Selenium and Its Association with Diet and Indoor Air Pollution among Pregnant Women in a Rural Area of Northern China

**DOI:** 10.3390/ijerph182212090

**Published:** 2021-11-18

**Authors:** Jiahe Liu, Lei Jin, Aiguo Ren

**Affiliations:** Institute of Reproductive and Child Health, National Health Commission Key Laboratory, Department of Epidemiology and Biostatistics, School of Public Health, Peking University, Beijing 100191, China; 1510306128@pku.edu.cn (J.L.); renag@pku.edu.cn (A.R.)

**Keywords:** selenium, pregnant women, China, food habits, indoor air pollution

## Abstract

The nutritional status of selenium (Se) in pregnant women in rural areas of northern China and its association with diet and indoor air pollution are rarely reported. We recruited 273 pregnant women in early or middle term in Shanxi and Hebei province and detected their fasting blood selenium. Demographic characteristics, food habits, and indoor air pollution exposure were collected with a questionnaire. Multivariate logistic regression models were used to estimate the adjusted odds ratios (AORs) and their 95% confidence intervals for the factors and relatively low blood levels of Se (below the lower quartile). The median (interquartile range [IQR]) blood concentration of Se was 117.35 (103.90, 129.23) μg/L. The rate of Se deficiency was 4.8%, and the rate of overnutrition was 23.8%. The AORs for the risk for relatively low blood levels of Se were 2.26 (1.15, 4.44) for consuming less beef and pork/mutton; 0.39 (0.19, 0.80) for a lower frequency of vinegar consumption; and 1.41 (0.76, 2.60) and 1.18 (0.59, 2.36) for passive smoking and indoor coal pollution, respectively. In conclusion, the nutritional status of Se in pregnant women in a rural area of northern China was acceptable; diet was the main determinant; no conclusive association was found between indoor air pollution and Se nutritional status.

## 1. Introduction

Selenium (Se) is an essential trace element in the human body [1]. It is transferred to the fetus through the placenta and plays an important role in pregnancy [2]. Several epidemiological studies have shown that low levels of Se in pregnancy may be associated with miscarriage, preterm birth, neural tube defects (NTDs), preeclampsia, and other maternal gestational complications [3,4,5,6,7], as well as with impact on the cognitive function of the infant [8]. The importance of Se as an essential trace element is linked to its composition of selenoproteins [9]. Selenoproteins are vital to maternal and infant health, and they contribute to antioxidation, immunoregulation, and antagonism [10]. The demand for Se significantly increases during gestation [2], and with the development of pregnancy, Se levels in serum gradually decline due to the active transport of Se to the placenta and hemodilution [11]. There is evidence to suggest that suboptimal Se intake during pregnancy is a common phenomenon [12].

China has one of the widest ranges and the most extensive degrees of Se deficiency in its geographical environment worldwide; a total of 51% of its territory suffers from Se deficiency [13]. The environmental levels of Se in most regions of North China are deficient or marginally deficient [14]. Shanxi and Hebei province once had the highest prevalence of NTDs in China. Although the prevalence of NTDs dropped significantly following the implementation of the maternal periconceptional folic acid supplement program, the rate remains high in Shanxi [15], which may be related to low levels of Se in local women [16]. The areas of serious Se deficiency are distributed in a belt from the northeast to the southwest of China [13]. Most of Shanxi and Hebei provinces are situated in northern China, adjacent to the Se deficiency belt and belonging to the marginal Se zone [14]; the same applies to three project counties. However, reports on Se status in pregnant women in areas with moderate Se deficiency in northern China have been relatively rare, and related factors have not been clearly described.

Selenium levels in the human body are greatly influenced by ecological conditions and dietary habits [1]. Environmental Se is enriched in the human body from the food chain [13], and it can also be concentrated through environmental conditions, such as coal combustion [17]. People living in rural areas in China are more likely to consume locally produced crops and meat than urban residents, which implies that rural women will be more sensitive to local environmental Se deficiencies than urban women. Coal is the most important fuel used for cooking and heating at home in northern China [18]. Most families operate a coal stove in their main rooms for heating or cooking in winter, which often causes the indoor air to be polluted with soot. However, it has not been established whether ambient Se from coal combustion for cooking and heating is a source of Se in blood in this area. Further, exposure to domestic passive smoking is a common phenomenon among women in rural China [19], and it may affect blood concentrations of Se in pregnant women [20].

Based on these considerations, in this study, healthy pregnant women were recruited from Daixian and Yushe counties in Shanxi Province and Xianghe county in Hebei Province to investigate their fasting blood levels of Se and to evaluate the nutritional status of Se in pregnant women in rural northern China in their first and second trimester of pregnancy, particularly in the vast area of low Se levels. We also investigated the relationship between the level of Se in pregnant women and dietary habits, as well as indoor air pollution, in the three selected counties.

## 2. Materials and Methods

### 2.1. Study Population

Three typical rural counties were selected for the survey. Yushe county is located in the south of Shanxi Province, and Daixian is located in its north. Xianghe county is located in the middle of Hebei Province. The samples included in this study offer a certain distributive representativeness for pregnant women in the vast rural areas with relatively low levels of environmental Se in northern China.

### 2.2. Study Participants

A total of 273 pregnant women in the counties were recruited from June to September 2010 by the medical staff of the local maternal and child healthcare hospital. The number of births in the three project counties was 6000–7000 per year. We recruited about 15% of pregnant women in the counties during the implementation of the project. As maternal and child health hospitals are the main institutions through which rural women receive healthcare during pregnancy, the sample is well representative of local women. Volunteers in the first or second trimester of pregnancy who had lived in any of the three counties for over 1 year at the time of the survey were included as participants. Those who had possible exposure to heavy metal pollution, such as through work in mining, the petroleum industry, or the paint industry, were excluded from the survey.

### 2.3. Questionnaire Survey

The subject enrollment, interviews, and blood collection were conducted by the doctors and nurses at the local maternal and child health hospital, who had received prior training. A face-to-face questionnaire interview approach was adopted to collect the demographic characteristics, dietary intakes, and environmental exposure of the participants. The sociodemographic characteristics collected included the following information: county of residence, age, occupation, education, household income, history of pregnancy, and childbirth. A self-developed food frequency questionnaire was adopted to investigate the consumption of fresh vegetables, pickled vegetables, beef, pork and mutton, poultry, aquatic products, dairy products, and so on. The FFQ questionnaire of the China Health and nutrition survey (CHNS) by the Chinese Center for Disease Control and Prevention was referred to when formulating the questionnaire. Since Shanxi is the main producing area of vinegar in China, and the residents of northern China customarily season food with vinegar, vinegar consumption was also included in the survey. We also collected information on forms of environmental exposure, such as passive smoking exposure, domestic fuel consumption, cooking habits, household patterns, occupational exposure, and the pollution status around the residence.

### 2.4. Blood Sample Collection

Fasting venous blood samples were collected from the women using 4 mL heparin lithium anticoagulant vacuum blood collection tubes (Becton, Dickinson and Company, BD, Sparks, MD, USA). After collection, the blood samples were frozen at −20 °C in the laboratory of the county maternal and child health hospital. Within a month of collection, the samples were transported on dry ice to Peking University Health Science Center for subsequent analyses.

### 2.5. Blood Se Determination

The concentration of Se was determined using inductively coupled plasma mass spectrometry (ICP-MS, 2 Perkin-Elmer Sciex Elan DRC II, Waltham, MA, USA) in the bio-metallomics laboratory of the Center of Medical and Health Analysis of Peking University. The sample pretreatment, detection, and quality control are detailed in a previous report [21]. Certified standards from Chinese national reference materials GSB 04-1751-2004 for Se were utilized for the calibration and validation of the standard curves. The regression lines for the calibration curves had correlation coefficients > 0.999. A sample of standard materials (GBW08552, derived from pig muscle) with known reference concentrations of Se was prepared and carried through the preparation and assay process alongside each of the twenty blood samples to ensure the stability of the process. The detection limit of ICP-MS for Se was 0.05 µg/L.

### 2.6. Se Nutritional Status Determination

Se nutritional status was determined with reference to an interval for blood concentrations of Se (90–130 μg/L) from the study of Reference Man by the International Commission on Radiological Protection (ICRP) [22], which is a general standard based on a number of inspection data from over 40 countries around the world, including the USA, Canada, China, Australia, Egypt, Finland, etc., [23]. Because no reference interval was found for pregnant women in particular, the Reference Man’s interval limits were used as the criterion to determine maternal Se levels in this study. The biomonitoring equivalent for whole blood related to Se toxicity is 400–480 μg/L [24], which is well above the maximum blood concentration of the samples in this study, so selenosis is beyond the scope of our study.

### 2.7. Definition of Exposure to Indoor Air Pollution

The specific questions relating to exposure to coal pollution in the questionnaire are reported in a previous study [25]. Exposure to coal pollution was quantified in three dimensions, and we assigned an exposure value for each dimension according to the responses to these questions. This definition is generally consistent with that of the previous study, except that the restriction on pregnancy during the heating season was relaxed when calculating the overall heat exposure. Se in plasma has a half-life of 233 ± 58 days [26]; there are grounds to believe that its half-life in whole blood is similar, so the samples collected in summer likely still reflected women’s exposure to Se from coal burning during the heating season. We created an index by summing the exposure values for all individual indoor coal pollution sources, as summarized below.

(1) Cooking exposure: When the primary home cooking fuel used was coal, kitchen exposure was recorded as 2 if the woman cooked daily, or as 1 if the woman cooked occasionally; otherwise, it was recorded as 0.
(2) Attached kitchen exposure: When the primary cooking fuel was coal and the kitchen was not separated from the living room or bedroom, the value was recorded as 1; otherwise, it was recorded as 0.
(3) Heating exposure: When the primary heating fuel was coal, and a coal stove was used for heating in the living room or bedroom, the value was recorded as 2 if the house was almost never ventilated, or as 1 if the house was occasionally ventilated; otherwise, the value was recorded as 0.

Exposure to indoor coal pollution was accorded one of two possible values; those for which the total index was ≥1 were defined as exposure to indoor coal pollution; otherwise, the result was no exposure.

### 2.8. Statistical Analyses

Because of the non-normal distribution of the data, the distribution of the whole-blood concentrations of Se was described using the median (interquartile range, IQR). Because the records fit better to a log-normal distribution, geometric means and 95% confidence intervals (CIs) are reported; the blood concentrations of Se were log-transformed (base 10) for use in multiple regression analyses. Chi square analyses were performed to determine whether the abnormal rate varied among different groups. Non-parametric analyses were used to compare the median levels of blood concentrations of Se between women with different characteristics. The Mann–Whitney test was conducted to compare the two groups, while the Kruskal–Wallis test was used to compare three or more groups.

Because the number of subjects with a strictly low blood level of Se was fairly small (4.8%), values lower than the 25% quantile were defined as relatively deficient Se levels in blood in subsequent analyses. Univariate and multivariate unconditional logistic regression models were used to estimate the factors associated with relatively low blood concentrations of Se. The strength of association is expressed using crude OR (cOR), adjusted OR (aOR), and their 95% CIs. The explanatory variables included in the model were as follows: province of residence; age group; education state; occupation; monthly per capita disposable income of family; gestational weeks; consumption frequency of aquatic products, poultry, beef, pork/mutton, pickled vegetable, vinegar, and dairy products; passive smoking; and indoor coal pollution. Each dietary variable other than vinegar consumption frequency was divided into two groups based on greater than or equal to once a week or less than once a week. As a frequently used condiment, vinegar consumption was divided based on greater than or equal to once a day or less than once a day.

The statistical analyses were performed using SPSS version 22.0 for Windows (SPSS Inc., Chicago, IL, USA). A two-sided test was employed, and *p* < 0.05 was considered statistically significant.

### 2.9. Ethical Statement

This study was conducted according to the guidelines laid down in the Declaration of Helsinki and all procedures involving the research study participants were approved by the institutional review board of Peking University. Written informed consent was obtained from the pregnant women. The ethical code of the study was IRB00001052-10106.

## 3. Results

### 3.1. Demographical Characteristics of the Study Participants

In all, 273 pregnant women were recruited for the survey, including 58 (21.2%) from Xianghe county, Hebei Province; 108 (39.6%) from Yushe county, Shanxi Province; and 107 (39.2%) from Daixian county, Shanxi Province. The age of the subjects ranged from 17 to 43 years, with an average age of (26.45 ± 5.28) years. Almost all of the participants, 271 (99.3%), were of Han ethnicity; 207 (75.5%) had education below high school; and 214 (78.4%) were farmers. In all, 169 (62.1%) of the subjects had a monthly disposable income of less than 500 yuan.

### 3.2. Se Concentration in Blood and Se Nutritional Status

The geometric mean of blood concentrations of Se in pregnant women was 116.81 (95% CI: 114.55, 119.13) μg/L, the median (IQR) was 117.35 (103.90, 129.23) μg/L, and the minimum value was 74.31 μg/L; the maximum value was 222.41 μg/L.

The median values (IQRs) of Se in blood among pregnant women in Yushe county, Daixian county, and Xianghe county were 112.65 (103.35, 119.04), 113.94 (102.32, 127.32), and 125.99 (114.20, 140.71) μg/L, respectively. They were 117.53 (107.48, 130.76) μg/L for women in the first trimester and 116.89 (101.10, 128.78) μg/L for women in the second trimester.

The rate of Se deficiency among the subjects in the study was 4.8%, and the overnutrition rate was 23.8%. The residents of Shanxi Province demonstrated a relatively high rate of deficiency (6.0%), and those of Hebei Province demonstrated a higher prevalence of Se overnutrition (31.0%), but the differences were not significant in either case. The differences in rates by gestational trimester also showed no statistical significance (Table 1).

### 3.3. Blood Concentrations of Se and Associated Factors

There were significant differences in blood Se concentrations among pregnant women in each county, and the levels were significantly lower in Shanxi Province than in Hebei Province (*p* < 0.05). Peasants appeared to have lower levels than other occupational groups, as did members of low-income families versus those from higher-income families. No statistically significant differences in blood concentrations of Se were observed among pregnant women at different ages, educational levels, gestational weeks, or gravidity. Among dietary habits, women who consumed more seafood, poultry, or beef and pork/mutton had higher Se concentrations, while those who had a preference for pickled vegetables and vinegar had lower concentrations. The Se levels among pregnant women with indoor coal pollution and exposure to passive smoking during pregnancy were lower than those without exposure. A multivariate log-linear regression model showed that only a high frequency of vinegar consumption was negatively associated with Se concentrations in whole blood (Table 2).

After adjusting for potential confounding factors in multivariate logistic models, the adjusted ORs for lower frequency of beef and pork/mutton and lower frequency of vinegar consumption were 2.26 (95% CI, 1.15, 4.44) and 0.39 (95% CI, 0.19, 0.80), respectively (Table 3).

## 4. Discussion

Using the interval from the Reference Man (90–130 μg/L), the rate of normal Se levels among pregnant women in the three counties was 71.4%, the rate of Se deficiency was 4.8%, and the rate of Se overnutrition was 23.8%; thus, the Se nutrition of the majority of pregnant women was in the normal range.

The maternal blood concentrations of Se in this study (Median (IQR): 117.35 (103.90, 129.23) μg/L) were clearly lower than those reported for Jiangsu Province (Median (IQR): 131.5 (114.0, 167.2) μg/L) [27], which is located in a moderate-Se ecological area; the same results were obtained in a study in Hubei Province (Median: 119.4 μg/L) [28]. Jiangsu Province is located on the eastern coast of China, and it features a high level of economy and urbanization. Hubei Province is located in central China; except for Enshi Prefecture, which is rich in Se, most areas of Hubei Province feature a moderate or low level of environmental Se. The levels of Se found in this study were higher than those in the UK (Median (IQR): 103.4 (75.0, 262.9) μg/L) [8] and far lower than those in Japan (Median (IQR): 178.0 (165.0, 192.0) μg/L) [29]. Se levels around the world vary widely. The ecological values of Se have a decisive impact on the nutritional level of Se in local residents. The three counties selected for the project are located in a marginal-Se zone, adjacent to the Se-deficiency belt, so the values found in these locations were lower than those found in Jiangsu. Europe is commonly considered to be an Se-deficiency area [30], and Se intake in Japan is generally higher than the values found in China, the UK, and the US [31]. Our conclusions are in line with this consensus.

Multivariate models suggested that dietary intake plays a vital role in maintaining the nutritional balance of Se. Although a relatively high frequency of consumption of beef and pork/mutton seemed to have no association with levels of Se, this may have been due to an insufficient sample size or simplistic survey methods rather than reflecting the actual influence, since our research also indicated that consuming beef and pork/mutton over once a week was a protective factor against low Se levels. We unexpectedly observed a significant association between higher-frequency consumption of vinegar and low blood levels of Se, which has not been reported previously. Among natural foods, animal viscera and seafood are the best sources of Se, followed by muscle meat, then cereals and dairy products, and finally vegetables and fruits, which include minimal levels of Se [32]; this explains the association between beef and pork/mutton and blood levels of Se. In China, rural women consume less meat than urban women [33], which means that under the same geographical conditions, rural women’s Se deficiency is more severe. Yushe and Daixian were listed as state-level poor counties at the time of the survey, and the nutrition of rural residents was not ideal. In our study, 40.7% of the participants ate meat less than once a week, which was very unfavorable for meeting the selenium intake requirements during pregnancy. Traditional Chinese vinegar is made from grain crops containing sugar or starch as its main raw material, and acetic acid is produced through secondary fermentation. The final product is rich in organic acids, amino acids, trace elements, and other substances, and it has been proven to have a variety of positive health functions [34]. The strong adverse association of vinegar consumption on maternal Se nutritional status in this study is not consistent with previous understanding. We originally thought that this result was related to the eating habits of Shanxi residents, who have a special preference for vinegar, but the results of the analyses of samples from Shanxi showed that the correlation remained significant (*p* < 0.05) (Appendix A). Limited by the level of education of rural women, we could not make an accurate estimation of vinegar consumption, which may cause misclassification. No adverse effects of vinegar on Se nutrition have been reported; therefore, further investigations are needed to determine the correlation.

An excellent source of Se, aquatic products showed no clear association with blood levels of Se. This is probably due to the traditional dietary structure in the inland counties of North China. The fish consumption of residents in Shanxi and Hebei provinces is in the lowest quartile of the country [35].

We did not find any impact of indoor ambience air pollution on the blood levels of Se among pregnant women in the selected counties. Se from coal combustion can induce Se poisoning [36]. More than 75% of the Se in coal volatilizes during high-temperature combustion, becoming easier to inhale or ingest, which has led to selenosis cases in Enshi, Hubei Province [17]. Shanxi is China’s largest producer of coal, and many households in rural areas use coal as a primary source for cooking and heating. Further, women generally perform most of the housework and thus are at the highest risk of exposure to coal pollution. Although our survey was conducted outside of the traditional heating season (November to March) in northern China, due to the long half-life of Se in blood [26], we would expect to continue to see the effects of Se pollution in indoor air caused by coal combustion. The non-significant relationship may be the result of the moderate level of Se content (2–5 mg/kg) in the coal of Shanxi Province [37].

The indispensability of Se in vivo is attributed to its role in reducing oxidative stress and the inhibition of apoptosis [38]. It has been shown that the Se levels of pregnant women exposed to passive smoking are low [27] because selenoproteins are consumed in oxidative stress as antioxidants [39]. However, we did not find a similar phenomenon in pregnant women with passive smoking exposure, which may have been due to the limitations of the sample size and/or survey precision. A negative association was demonstrated between Se and age in the general population [40,41]. However, partly because of the narrow age range (17–43) of the pregnant women recruited, and the limitations of the sample size, we did not find a similar correlation among pregnant women.

Few studies on the nutritional status of Se have been conducted among pregnant women in a rural, northern area of China. Whole-blood Se concentrations were used to evaluate the nutritional status of Se in pregnant women in the early or mid-gestational period, a group seldom examined in previous studies. Diet, indoor air pollution, and demographic characteristics were considered to be possibly associated with the nutritional status of Se or risk for lower blood levels of Se. However, our study featured certain limitations. A food-frequency questionnaire was used, which could not measure the exact amount of Se intake; the sample size was not large, particularly for pregnant women in Hebei Province.

## 5. Conclusions

In summary, Se levels in pregnant women at the first and second trimester of the gestational period were found to be at an acceptable level in the three northern rural counties considered. Diet was the main determinant of the level of Se in pregnant women. No conclusive association between indoor air pollution and Se nutrition in pregnant women was found.

## Figures and Tables

**Table 1:** Prevalence of Se deficiency and overnutrition among pregnant women in three counties of northern China.

Characteristic	Total *n*	Se Deficiency (< 90 µg/L)			Se Overnutrition (>130 µg/L)		
		*n* (%)	*χ* ^2^	*p*	*n* (%)	*χ* ^2^	*p*
Total	273	13 (4.8)			65 (23.8)		
County							
Yushe	108	5 (4.6)	4.64	0.098	26 (24.1)	2.71	0.259
Dai	107	8 (7.5)			21 (19.6)		
Xianghe	58	0			18 (31.0)		
Province							
Shanxi	215	13 (6.0)	3.68	0.055	47 (21.9)	2.12	0.145
Hebei	58	0			18 (31.0)		
Gestational weeks							
4–13	96	2 (2.1)	2.34	0.126	26 (27.1)	0.88	0.350
13–28	177	11 (6.2)			39 (22.0)		

**Table 2:** Se concentrations in blood in pregnant women and associated factors.

Characteristic	*n* ^a^ (%)	Non-Parametric Tests		Log-Linear Regression Model ^d^		
		Median (IQR)/(μg/L)	*p* ^b^	*β*	*SE*	*p*
County			0.001			
Yushe	108 (39.6)	112.65 (103.35, 119.04)		-		
Dai	107 (39.2)	113.94 (102.32, 127.32)				
Xianghe	58 (21.2)	125.99 (114.20, 140.71)				
Province			<0.001			
Shanxi	215 (78.8)	112.96 (102.57, 127.97)		−0.015	0.016	0.343
Hebei	58 (21.2)	125.99 (114.20, 140.71)		0 ^c^		
Age			0.581			
17–25	115 (42.1)	118.69 (105.90, 127.96)		−0.001	0.013	0.929
25–30	83 (30.8)	117.71 (104.62, 131.78)		0.005	0.012	0.696
30–43	74 (27.1)	114.05 (100.92, 130.66)		0 ^c^		
Education			0.251			
Junior high school or less	207 (75.5)	117.35 (103.31, 128.59)		−0.003	0.012	0.806
High school or more	65 (24.5)	117.35 (105.86, 133.83)		0 ^c^		
Occupation			0.017			
Farmer	214 (78.4)	114.75 (103.52, 127.90)		−0.011	0.013	0.417
Others	58 (21.6)	123.22 (109.13, 136.61)		0 ^c^		
Per capita disposable cash of family (yuan/month)			0.015			
<500	169 (62.1)	113.94 (102.45, 128.26)		−0.008	0.011	0.475
≥500	103 (37.9)	119.85 (106.20, 131.39)		0 ^c^		
Gestational week/weeks			0.162			
1–13	96 (35.2)	117.53 (107.48, 130.76)		0.004	0.009	0.674
14–28	177 (64.8)	116.89 (101.10, 128.78)		0 ^c^		
Spontaneous abortion			0.521			
Yes	70 (25.9)	115.51 (104.74, 128.57)		−0.008	0.011	0.445
No	200 (74.1)	118.23 (104.05, 129.83)		0 ^c^		
Gravidity			0.686			
1	102 (37.6)	119.03 (104.59, 128.52)		−0.001	0.012	0.960
≥2	169 (62.4)	116.02 (103.87, 130.39)		0 ^c^		
Aquatic products			0.071			
<1 time/week	222 (81.3)	115.99 (103.90, 128.77)		0.007	0.013	0.572
≥1 time/week	51 (18.7)	121.83 (102.87, 130.09)		0 ^c^		
Poultry			0.004			
<1 time/week	181 (66.5)	112.85 (102.54, 128.04)		−0.002	0.010	0.878
≥1 time/week	91 (33.5)	121.83 (111.03, 131.41)		0 ^c^		
Beef, pork, and mutton			0.008			
<1 time/week	111 (40.7)	112.45 (100.20, 126.54)		−0.015	0.009	0.112
≥1 time/week	162 (59.3)	118.96 (106.04, 131.02)		0 ^c^		
Pickled vegetables			0.001			
<1 time/week	170 (62.7)	119.71 (107.53, 131.49)		0.011	0.009	0.209
≥1 time/week	101 (37.3)	111.21 (99.90, 126.60)		0 ^c^		
Dairy products			0.983			
<1 time/week	138 (50.5)	117.35 (103.78, 129.23)		0.010	0.009	0.263
≥1 time/week	135 (49.5)	116.98 (105.12, 129.27)		0 ^c^		
Vinegar			0.000			
<1 time/day	136 (50.0)	122.61 (109.29, 136.15)		0.029	0.010	0.003
≥1 time/day	136 (50.0)	110.89 (100.74, 124.54)		0 ^c^		
Exposure to passive smoking			0.047			
Yes	134 (49.1)	112.95 (103.10, 127.42)		−0.016	0.008	0.056
No	139 (50.9)	119.20 (105.79, 132.54)		0 ^c^		
Exposure to indoor coal pollution			0.049			
Yes	142 (52.0)	115.47 (102.46, 128.50)		−0.009	0.010	0.331
No	131 (48.0)	118.72 (105.90, 130.09)		0 ^c^		

^a^ Due to missing data, the sum of the number of subjects in each group may be less than 273. ^b^ *p* for non-parametric tests, Mann–Whitney U test was conducted for comparison between two groups, while the Kruskal–Wallis test was used for comparison of three or more groups. ^c^ Set to zero because this parameter is redundant. ^d^ The blood concentrations of Se were log-transformed (base 10) for multivariate regression model analyses.

**Table 3:** Factors associated with relatively lower blood levels of Se among pregnant women in the first or second trimesters.

Characteristic	*n* ^a^	Low Blood Se ^b^, *n* (%)	COR (95% CI)	AOR (95% CI)
Province				
Shanxi	214	60 (28.0)	2.89 (1.24, 6.71)	1.49 (0.40, 5.55)
Hebei	56	6 (10.7)	1	1
Age				
17–25	113	22 (19.5)	0.52 (0.27, 1.02)	0.53 (0.24, 1.18)
25–30	83	20 (24.1)	0.66 (0.33, 1.33)	0.70 (0.31, 1.57)
30–43	74	24 (32.4)	1	1
Education				
Junior high school or lower	205	54 (26.3)	1.60 (0.80, 3.21)	1.48 (0.59, 3.75)
High school and above	65	12 (18.5)	1	1
Occupation				
Farmer	212	56 (26.4)	1.74 (0.83, 3.67)	1.07 (0.39, 2.93)
Others	58	10 (17.2)	1	1
Per capita disposable cash of family (yuan/month)				
<500	169	47 (27.8)	1.60 (0.88, 2.89)	0.86 (0.39, 1.90)
≥500	101	19 (18.8)	1	1
Gestational week/weeks				
1–13	95	17 (17.9)	0.53 (0.29, 0.99)	0.67 (0.34, 1.32)
14–28	175	49 (28.0)	1	1
Aquatic products				
<1 time/week	222	55 (24.8)	1.04 (0.52, 2.09)	0.53 (0.21, 1.37)
≥1 time/week	48	11 (22.9)	1	1
Poultry				
<1 time/week	180	50 (27.8)	1.84 (0.98, 3.45)	0.80 (0.36, 1.77)
≥1 time/week	90	16 (17.8)	1	1
Beef, pork, and mutton				
<1 time/week	111	37 (33.3)	2.11 (1.21, 3.68)	2.26 (1.15, 4.44)
≥1 time/week	159	29 (18.2)	1	1
Pickled vegetables				
<1 time/week	170	31 (18.2)	0.40 (0.23, 0.71)	0.60 (0.32, 1.11)
≥1 time/week	100	35 (35.0)	1	1
Vinegar				
<1 time/day	135	20 (14.8)	0.35 (0.19, 0.62)	0.39 (0.19, 0.80)
≥1 time/day	135	46 (34.1)		1
Dairy products				
<1 time/week	137	35 (25.5)	1.14 (0.66, 1.97)	0.86 (0.45, 1.64)
≥1 time/week	133	31 (23.3)		1
Passive smoker				
Yes	136	38 (27.9)	1.44 (0.83, 2.50)	1.41 (0.76, 2.60)
No	134	28 (20.9)	1	1
Exposure to indoor coal pollution				
Yes	140	40 (28.6)	1.56 (0.90, 2.73)	1.18 (0.59, 2.36)
No	130	26 (20.0)	1	1

^a^ Due to missing data, the sum of the number of subjects in each group may be less than 273. ^b^ Lower than the 25% percentile of all samples is defined as low blood levels of Se.

## Data Availability

Data is contained within the article.

## Appendix A

**Table A1:** Factors associated with low levels of Se in blood among pregnant women in a rural area of Shanxi.

Characteristic	*n*	Relative Low Blood Se ^a^, *n* (%)	AOR (95% CI)
Age			
17–25	76	20 (26.3)	0.54 (0.23, 1.26)
25–30	71	16 (22.5)	0.50 (0.21, 1.22)
30–43	67	24 (35.8)	1
Education			
Lower than junior high school	160	49 (30.6)	1.63 (0.60, 4.42)
High school and above	54	11 (20.4)	1
Occupation			
Farmer	178	53 (29.8)	1.13 (0.35, 3.65)
Others	36	7 (19.4)	1
Per capita disposable cash of family (yuan/month)			
<500	159	47 (29.6)	0.85 (0.36, 2.02)
≥500	55	13 (23.6)	1
Gestational week/weeks			
1–13	62	12 (19.4)	0.47 (0.21, 1.02)
14–28	152	49 (32.2)	1
Aquatic products			
<1 time/week	196	52 (26.5)	0.37 (0.12, 1.13)
≥1 time/week	18	9 (50.0)	1
Poultry			
<1 time/week	156	49 (31.4)	0.85 (0.35, 2.11)
≥1 time/week	58	12 (20.7)	1
Beef, pork, and mutton			
<1 time/week	103	36 (35.0)	2.25 (1.19, 5.23)
≥1 time/week	111	25 (22.5)	1
Pickled vegetables			
<1 time/week	122	28 (22.9)	0.73 (0.38, 1.42)
≥1 time/week	92	33 (35.9)	1
Vinegar			
<1 time/day	84	15 (17.9)	0.37 (0.17, 0.81)
≥1 time/day	130	46 (35.4)	1
Dairy products			
<1 time/week	114	32 (28.1)	0.77 (0.39, 1.54)
≥1 time/week	100	29 (29.0)	1
Passive smoke			
Yes	114	35 (30.7)	1.28 (0.66, 2.46)
No	100	26 (26.0)	1
Exposure to indoor coal pollution			
Yes	126	39 (31.0)	1.30 (0.61, 2.75)
No	88	22 (25.0)	1

^a^ The value within the lowest quartile of all samples is defined as low blood levels of Se.
